# E2F1 mediates the downregulation of POLD1 in replicative senescence

**DOI:** 10.1007/s00018-019-03070-z

**Published:** 2019-03-20

**Authors:** Shichao Gao, Qiao Song, Jing Liu, Xiaomin Zhang, Xunming Ji, Peichang Wang

**Affiliations:** 1 Clinical Laboratory of Xuanwu Hospital, Capital Medical University, Beijing, 100053 People’s Republic of China; 2 Beijing Institute of Brain Disorders, Capital Medical University, Beijing, 100053 People’s Republic of China

**Keywords:** POLD1, Transcription factor, E2F1, DNA methylation, Replicative senescence

## Abstract

POLD1, the catalytic subunit of DNA Pol δ, plays an important role in DNA synthesis and DNA damage repair, and POLD1 is downregulated in replicative senescence and mediates cell aging. However, the mechanisms of age-related downregulation of POLD1 expression have not been elucidated. In this study, four potential CpG islands in the POLD1 promoter were found, and the methylation levels of the POLD1 promoter were increased in aging 2BS cells, WI-38 cells and peripheral blood lymphocytes, especially at a single site, CpG 36, in CpG island 3. Then, the transcription factor E2F1 was observed to bind to these sites. The binding affinity of E2F1 for the POLD1 promoter was found to show age-related attenuation and was confirmed to be positively regulated by the E2F1 level and negatively regulated by POLD1 promoter methylation. Moreover, cell senescence characteristics were observed in the cells transfected with shRNA-E2F1 and could contribute to the downregulation of POLD1 induced by the E2F1 decline. Collectively, these results indicated that the attenuation of the binding affinity of E2F1 for the POLD1 promoter, mediated by an age-related decline in E2F1 and increased methylation of CpG island 3, downregulates POLD1 expression in aging.

## Introduction

Cellular senescence is a cellular state that occurs after serial cultivation, with subsequent irreversible growth arrest and loss of proliferative ability [1]. Senescence is characterized by a series of particular changes in cellular morphology and senescence-associated genes [2]. Incomplete maintenance of genomic integrity is considered to be an important cause of cell senescence and premature aging [3].

DNA polymerases play an essential role in the maintenance of genome integrity. The DNA polymerase delta (Pol δ) functions in DNA synthesis and DNA damage repair, which are important for cell proliferation [4]. The catalytic subunit of DNA Pol δ (p125) is encoded by the POLD1 gene [5]. Our previous study found that POLD1 expression was downregulated with cell aging and tentatively confirmed that the downregulation of POLD1 could be essential in senescence-related processes [6]. The age-related decline in POLD1 causes senescence by reducing DNA synthesis and DNA damage repair [7]. However, the underlying mechanism of POLD1 deregulation during cellular senescence is not clear.

The POLD1 promoter is rich in GC dinucleotides, does not contain a TATA box, and has many potential transcription factor (TF) binding sites [8]. Based on the structural characteristics of the POLD1 promoter region, we speculated that key transcriptional factors could bind to the POLD1 promoter and regulate POLD1 expression.

DNA methylation of CpG dinucleotides in gene promoters plays an important role in gene expression regulation [9]. In general, the demethylation of CpG islands in gene regulatory elements indicates transcriptional activity, whereas methylation of cytosines in CpG islands results in gene silencing [10]. Age-related CpG island hypermethylation in the promoter region usually leads to inhibition of the expression of related genes and may contribute to cellular senescence through a gene-associated reduction in cell proliferation [11].

There are many potential TF binding sites (TFBS) in the CpG islands, including the binding site for transcription factor E2F1, which has pivotal functions in the cell cycle, apoptosis and differentiation [12]. As an oncogene, E2F1 overexpression promotes cell progression from G0 to S phase [13]. E2F1 is overexpressed in HEL erythrocyte leukemia cells, and its upregulation can induce astrocyte tumorigenesis in vitro [14]. However, in other cases, E2F1 has the ability to induce p53-dependent and p53-independent apoptosis, suggesting tumor suppressive activity [15]. When E2F1 function is affected or when E2F1 DNA binding activity is blocked, cell growth is inhibited [16].

In this study, four potential CpG islands in the POLD1 promoter region were found, and E2F1 was predicted to bind to CpG island 3 of the POLD1 promoter. We hypothesized that the attenuation of the binding affinity of E2F1 for the POLD1 promoter, mediated by an age-related decline in E2F1 and an increase in CpG island 3 methylation, downregulated POLD1 expression in replicative senescence.

## Materials and methods

### Healthy donors

Healthy donors were recruited from a population of healthy people from the health examination center of Xuanwu hospital. After providing informed consent, each participant completed a brief questionnaire containing questions related to their health, tobacco consumption, and demographics. Each donor provided 5 ml of whole blood, extracted between 9 and 11 a.m. under nonfasting conditions. Donors with signs or a history of recent infection were excluded. One hundred participants, including women and men who were randomly selected, were enrolled in this study and divided into eight age groups: ages 20–30 years (*n* = 14, mean age of 24 ± 6.3 years), 30–40 years (*n* = 14, mean age of 35 ± 7.2 years), 40–50 years (*n* = 14, mean age of 44 ± 6.8 years), 50–60 years (*n* = 14, mean age of 54 ± 5.5 years), 60–70 years (*n* = 14, mean age of 66 ± 7.8 years), 70–80 years (*n* = 14, mean age of 74 ± 6.1 years), 80–90 years (*n* = 8, mean age of 85 ± 7.1 years) and over 90 years (*n* = 8, mean age of 93 ± 4.2 years).

### Lymphocyte isolation

Five milliliters of venous blood with the anticoagulant EDTA-K_2_ was drawn from the volunteers. Peripheral blood mononuclear cells (PBMCs) were obtained by Ficoll-Paque (Solarbio, Beijing, China) density gradient centrifugation. PBMCs, consisting mainly of lymphocytes, were used for a series of experiments.

### Bioinformatic analysis

The 2000-bp sequence upstream of the POLD1 transcript start site (TSS) was obtained from the Ensembl genome browser (http://www.ensembl.org/), and putative CpG islands were identified using MethPromer online tools (http://www.urogene.org/methprimer/). A CpG island was defined as a region of at least 100 bp, with more than 50% GC content, and an observed-to-expected (O/E) CpG ratio > 0.6. The DNA methylation patterns were analyzed using QUMA online tools (http://quma.cdb.riken.jp/). The TRANSFAC database (http://gene-regulation.com/) was used to predict the transcription factor binding sites of the POLD1 promoter. The potential E2F1 binding site sequence was TTTSSCGC (with S=C or G).

### Cell lines and culture conditions

Human embryonic lung diploid fibroblasts (2BS) were obtained from the Peking University Research Center of Aging (Beijing, China). Human fetal lung fibroblasts (WI-38) were purchased from the National Infrastructure of Cell Line Resource (Beijing, China). HEK293T cells were purchased from the Country Cell Bank, Shanghai Cell Institute (Shanghai, China). The 2BS, WI-38 and HEK293T cells were cultured in RPMI 1640 medium (GIBCO, Gaithersburg, MD, USA), minimum essential medium (MEM, GIBCO) and Dulbecco’s modified Eagle’s medium (DMEM, GIBCO), respectively. All the culture media were supplemented with 10% fetal bovine serum (FBS, GIBCO) and 100 U/ml penicillin and 100 µg/ml streptomycin, and the cells were then cultured in a humidified incubator with 5% CO_2_ at 37 °C. Cell cultures were expanded through sequential subculturing using trypsin–EDTA (GIBCO) to achieve a higher population doubling (PD) level. Cells grown to approximately 80–90% confluence were used for all experiments.

### 5-Aza-2′-deoxycytidine (5-AzaC) treatment

Middle-aged 2BS (48PD) and WI-38 (38PD) cells were plated at a density of 10^4^ cells/cm^2^ and treated with 5 μM 5-AzaC (Sigma, St Louis, MO, USA) 24 h later. The medium was changed every 24 h with medium containing fresh 5-AzaC, and after 72 h, the cells were collected for experiments.

### Overexpression lentivirus and shRNA

To construct a lentivirus for the overexpression of E2F1, the full-length E2F1 cDNA (GenBank accession number: NM_005225.2) was amplified by polymerase chain reaction (PCR) and subcloned into the pLenti-CMV vector to construct pLenti-CMV-E2F1. Then, the packaging cell line for the lentivirus vector was established. Positive clones were confirmed by DNA sequencing. Short hairpin RNA (shRNA) targeting E2F1 (shRNA-E2F1) and the negative control shRNA (shControl) were purchased from BioGot technology (Nanjing, China). The primers used are shown in Table 1.

**Table 1:** Primer sequence for PCR, real-time PCR, bisulfite sequencing analysis (BSP)

Primers for real-time quantitative RT-PCR		
POLD1	5′-CAACCTGGTCACTGCCTCAC-3′	5′-GTCCCGCTTCCTCATCCTCT-3′
E2F1	5′-ACTGAATCTGACCACCAAGCG-3′	5′-CAGGGTCTGCAATGCTACGA-3′
GAPDH	5′-CGAGTCAACGGATTTGGTGGTAT-3′	5′-AGCCTTCTCCATGGTGAAGAC-3′
Primers used for the generation of POLD1 promoter/reporter		
POLD1 P(− 1758/+ 49)	5′-CAGCCTTGCCTGACCACC-3′ (NheI)	5′-ACAGCGTTTCCCGCCACA-3′ (Hind III)
Primers used for E2F1 expression vector construct		
E2F1	5′-CGACCCTGACCTGCTGCTCT-3′ (EcoR I)	5′-TCTGATGCCCTCGCCCTCCT-3′ (BamH I)
shRNA designed to target E2F1 (Target sequence: 5′-GACGTGTCAGGACCTTCGTT-3′)		
E2F1	5′-TTGGATTTGGGATCTTAT-3′ (BamH I)	5′-TTGTGATGCTATTGCTTT-3′ (EcoR I)
Primers for ChIP assay promoter-specific PCR		
POLD1	5′-CTGTGGCGGGAAACGCTGTT-3′	5′-TCTATGTGCCTGGCTGCTCAAG-3′
β-Actin	5′-ACGCCAAAACTCTCCCTCCTCCTC-3′	5′-CATAAAAGGCAACTTTCGGAACGGC-3′
Primers used for bisulfite sequencing analysis		
CpG island1 first	5′-TAAAGTTTGGTAGGGTAGGGG-3′	5′-AATACCGACGTCTATCTAC-3′
CpG island1 second	5′-TTTTTTTAGGATTAGTTG-3′	5′-CTAAATAACCAACCCCTCACTC-3′
CpG island2 first	5′-GTGGGGTTAGAGGAAATTAG-3′	5′-TACATCCCTCCATCCAACCCC-3′
CpG island2 second	5′-GGGAAGGTATTATAATAGAGG-3′	5′-AATTACTCATTCAACTCCTTC-3′
CpG island3 first	5′-GAAATTGAAGTTAGGATGAAGG-3′	5′-ATACCTAACTACTCAAACCCC-3′
CpG island3 second	5′-GGAGAATAGATTAAGTTAGG-3′	5′-ATACCTAACTACTCAAACCCC-3′
CpG island4 first	5′-GTTTGAGTAGTTAGGTATATAG-3′	5′-CCCGAACCACRTCCCGCCCCAC-3′
CpG island4 second	5′-AGGTATATAGATTTGAGAGA-3′	5′-CCCCACGATACCCRACRCATAC-3′

### Lentivirus transfection

2BS and WI-38 cells were seeded in 24-well culture plates at a cell density of 1 × 10^5^/well 1 day before transfection. Then, overexpression lentivirus and shRNA as well as negative controls were transfected into 2BS and WI-38 cells using polybrene (Sigma, St Louis, MO, USA). At 48–72 h after transfection, the cells were collected for further analysis.

### Reverse transcription PCR (RT-PCR)

RNA was extracted using the RNeasy Mini Kit (Qiagen, Valencia, CA, USA) according to the manufacturer’s protocol. Reverse transcription reactions were performed using a ThermoScript™ RT-PCR System (Invitrogen, Carlsbad, CA, USA) and an oligo (dT) 20 primer. PCR amplification was carried out to measure the first strand cDNA of POLD1 and E2F1 mRNA, and GAPDH was used as the control. The PCR products were identified by agarose gel electrophoresis. The sequences of the primers are listed in Table 1.

### Protein extraction and western blot analysis

Cells were lysed in RIPA lysis buffer (Solarbio, Beijing, China) with protease inhibitor phenylmethanesulfonyl fluoride (PMSF, Beyotime, Nanjing, China). The protein concentration was determined using a BCA Protein Assay kit (Solarbio, Beijing, China). Equal amounts of protein were loaded onto SDS-PAGE gels (with 10% SDS) and separated by electrophoresis. The isolated proteins were then transferred to PVDF membranes (Millipore, California, USA). The membranes were blocked with 5% w/v nonfat milk for 1 h at room temperature. The membranes were then incubated with POLD1 antibodies (1:1000 dilution; Abcam, Cambridge, UK), E2F1 antibodies (1:1000 dilution; Abcam) and GAPDH antibodies (1:5000 dilution; Abcam) overnight at 4 °C. After incubation with the corresponding horseradish peroxidase (HRP)-conjugated secondary antibodies (ZSGB-BIO, Beijing, China), the protein bands were visualized using an Immobilon™ Western kit (Millipore). The bands were quantified with ImageJ software (National Institutes of Health, NIH, USA), and the target protein level was normalized to GAPDH.

### Extraction of genomic DNA and determination of global DNA methylation

Genomic DNA extraction was performed using the TIANamp Genomic DNA kit (Tiangen Biotech, Beijing, China), and global DNA methylation determination was performed using the Methylamp™ Global DNA methylation Quantification Kit (Epigentek Group, Farmingdale, NY, USA) according to the instructions provided by the manufacturer. The kit can accurately measure the percentage of methylcytosine in the total cytosine content.

### Bisulfite sequencing PCR (BSP)

Genomic DNA bisulfite conversion was performed using the Epitect Fast Bisulfite Kit (Qiagen, Valencia, CA, USA). The four CpG islands of the POLD1 promoter fragment were amplified by nested PCR using EpiTaq PCR buffer (Takara, Kyoto, Japan) and cloned into the T-vector pMD™19 (Takara). Ten independent clones were sequenced for each of the amplified fragments. The PCR primers are shown in Table 1.

### Patch methylation and luciferase activity assay

The whole length (− 1758 ~ + 49, 1808 bp) of the POLD1 promoter was cloned into the pGL3-Basic vector (Promega, Madison, WI, USA), named pGL3-Basic-POLD1 (primers used see Table 1). Twenty micrograms of pGL3-Basic-POLD1 was treated with 20 units of SssI methylase and 160 μM S-adenosylmethionine (SAM, NEB, Ipswich, MA, USA). The methylation status of pGL3-Basic-POLD1 was identified by methylation-sensitive restriction endonuclease Aci I (NEB) digestion. Controls included unmethylated constructs similarly generated but omitting the SssI. The methylated or unmethylated pGL3-POLD1 constructs were then transfected into HEK-293T cells; β-galactosidase luciferase expression constructs based on the pRL-CMV vector (Promega) were used as transfection controls, and the pGL3-Basic vector was used as the negative control. After 24 h, the cells were washed twice with PBS, and the luciferase or β-galactosidase levels were measured using the Dual-Luciferase Report kit (Promega).

### Chromatin immunoprecipitation (ChIP) assay

ChIP assays were carried out using the ChIP Assay kit (Abclonal, Woburn, MA, USA) according to the manufacturer’s protocol. Briefly, cells were crosslinked by formaldehyde treatment, followed by ultrasonication. Immunoprecipitation was carried out with 2 μg of anti-E2F1 antibody (Abcam, Cambridge, UK) at 4 °C overnight with rotation. The purified DNA was amplified with specific primers (see Table 1). The PCR products were analyzed by 1% agarose gel electrophoresis. Normal rabbit IgG was used as the negative control, and 5% input DNA was used as the positive control.

### Cell proliferation assay

The proliferation of 2BS and WI-38 cells was tested with the Cell Counting Kit-8 kit (CCK-8, Beyotime, Beijing, China). Briefly, cells were seeded in triplicate in 96-well plates at 5 × 10^4^ cells/well. At 0, 1, 3, and 6 days after treatment, 10 μl of CCK-8 solution was added to each well, and the plates were incubated at 37 °C for 2 h. The absorbance values of all wells at 450 nm were determined with an enzyme-labeling instrument (Thermo Fisher Scientific, Carlsbad, CA, USA).

### 5-Ethynyl-2′-deoxyuridine (EdU) incorporation assay

The DNA synthesis rate was assessed using the BeyoClick™ EdU kit (Beyotime, Beijing, China). The cells were incubated with 10 μM EdU for 2 h, followed by staining according to the manufacturer’s instructions. The absorbance values of all wells at 450 nm were determined with an enzyme-labeling instrument (Thermo Fisher Scientific, Carlsbad, CA, USA).

### Comet assay

Comet assays were used to examine 2BS and WI-38 cells against oxidative DNA damage under different conditions. At 48 h after transfection with E2F1 overexpression lentivirus/shRNA-E2F1, the cells were treated with 100 μM H_2_O_2_ for 5 min at 4 °C in the dark. Cells were then washed with PBS and immediately analyzed using a Comet Assay kit (Trevigen, Gaithersburg, MD, USA) according to the manufacturer’s protocol. A fluorescence microscope (OLYMPUS, Japan) was then used to observe the state of the cells. The olive tail moment (OTM) of each cell was analyzed using CASP software (version 1.2.3, download in http://casplab.com/).

### Statistical analysis

Statistical analysis was carried out using SPSS 23.0 software (Chicago, IL, USA). All data are presented as the mean ± SD (standard deviation). The difference between two groups was analyzed using a two-tailed *t* test. The differences among more than two groups were analyzed using one-way analysis of variance (ANOVA) followed by the least significance difference method (LSD) test for the chosen group. The CCK-8 data were analyzed using two-way ANOVA with repeated measures. Correlation analysis between the methylation level of the POLD1 promoter and POLD1 expression was examined using Pearson’s correlation coefficient. The correlation between E2F1 and POLD1 expression was calculated with Spearman’s rho method. *P* < 0.05 was considered significant.

## Results

### The alteration of methylation levels at the POLD1 promoter and the CpG islands in the promoter in the replicative senescence of 2BS and WI-38 cells

**Fig. 1:**
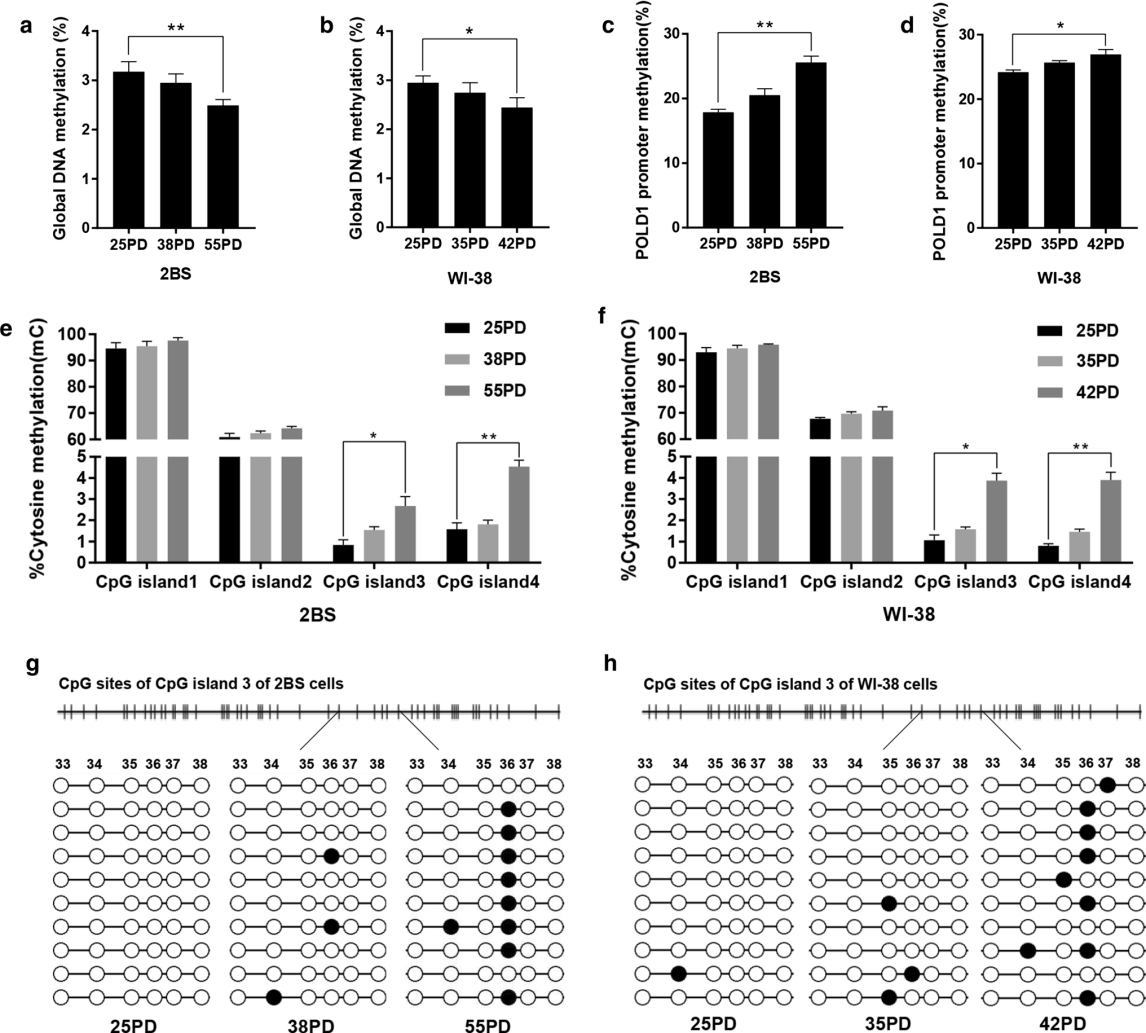
The global DNA and POLD1 promoter methylation levels in the replicative senescence of 2BS and WI-38 cells. **a**, **b** Global genome DNA methylation (%) in different PDs of 2BS and WI-38 cells. Global DNA methylation was measured using the Methylamp™ Global DNA methylation Quantification Kit. **c**, **d** DNA methylation status of CpG islands located in the region of the POLD1 promoter in different PDs of 2BS and WI-38 cells, measured by bisulfite DNA sequencing analysis. **e**, **f** The percentage of cytosine methylation of each CpG island of the POLD1 promoter in different PDs of 2BS and WI-38 cells. The data were analyzed by one-way ANOVA, three independent experiments in each group, *p < 0.05, **p < 0.01, vs. the young cells (25PD). **g**, **h** The DNA methylation pattern of part of CpG island 3 in the POLD1 promoter at different PDs of 2BS and WI-38 cells. The methylation levels of the sequence between the 33 CpG site and 38 CpG site and the methylation of the 36 CpG site increased significantly in replicative senescence. Empty circle, unmethylated cytosine; filled circle, and methylated cytosine

The global DNA and the POLD1 promoter DNA methylation levels were observed in different PDs of 2BS and WI-38 cells. The results showed that the global DNA methylation level decreased significantly with cell aging (Fig. 1a, b). However, the methylation level of the POLD1 promoter increased significantly in replicative senescence (Fig. 1c, d).

The CpG islands in the POLD1 promoter were predicted using MethPrimer online tools (http://www.urogene.org/methprimer/), and the methylation status of each CpG island of the POLD1 promoter was analyzed. The results showed that there were four CpG islands in the POLD1 promoter region: CpG island 1 (109 bp), located in the − 1878 to − 1770 region; CpG island 2 (102 bp), located in the − 767 to − 666 region; CpG island 3 (504 bp), located in the − 408 to + 95 region; and CpG islands 4 (100 bp), located in the + 147 to + 246 region. Then, bisulfite sequencing was used to identify the methylation patterns of the POLD1 promoter in different PDs of 2BS and WI-38 cells. Table 2 and Fig. 1c, d show the methylation status of each CG in the four CpG islands of the POLD1 promoter region in young, middle-aged, and senescent cells. Overall, the methylation status of the POLD1 promoter region increased significantly in aging cells compared to young cells.

**Table 2:** Bisulfite sequencing analysis of the CpG islands in the POLD1 promoter of different-aged 2BS and WI-38 cells

Cell	PD	Clones	Total CpGs	mCpGs	Ratio of mCpGs/total CpGs (%)
2BS	25	10	880	150	17.05
	38	10	880	168	19.09
	55	10	880	219	24.89
WI-38	25	10	880	213	24.20
	35	10	880	227	25.80
	42	10	880	241	27.39

In addition, the percentage of cytosine methylation of each CpG island located at the CpG region of the POLD1 promoter in different PDs of 2BS and WI-38 cells was analyzed. As shown in Fig. 1e, f, CpG islands 1 and 2 were hypermethylated, with no significant changes in replicative senescence. In contrast, CpG islands 3 and 4 were hypomethylated, but there was a significant increase in methylation as the cells aged.

The methylation changes at the CpG sites in the CpG islands were also analyzed, and the results showed that the methylation of a special single site, CpG 36, in CpG island 3, increased markedly with cell aging (Fig. 1g, h), which were very unique methylation changes.

### E2F1 binds to the POLD1 promoter and the binding site in the POLD1 promoter

**Fig. 2:**
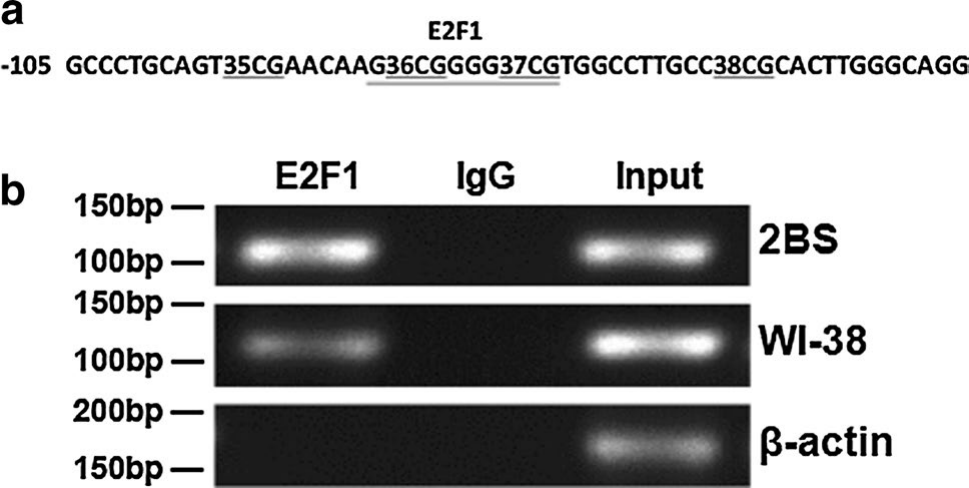
The binding of E2F1 to the POLD1 promoter in vivo. **a** Transcription factor analysis in CpG island 3 of the POLD1 promoter using the TRANSFAC database and showing a potential E2F1-binding site (underlined) in the 36 CpG sites of CpG island 3. **b** The binding of E2F1 to the POLD1 promoter was analyzed by ChIP assay in 2BS and WI-38 cells. Normal rabbit IgG was used as the negative control, and input was used as the positive amplification control, indicating 5% input DNA. The β-action test was used as the control to exclude false positives from genomic DNA pollution

The potential E2F1 binding sites were analyzed with the assistance of the TRANSFAC database, and the results showed a potential E2F1 binding site in the 36 CpG sites of the CpG island 3 (Fig. 2a).

To directly demonstrate that E2F1 binds to the putative binding site within CpG island 3 of the POLD1 promoter, ChIP assays were performed in 2BS and WI-38 cells. The ChIP results demonstrated that E2F1 bound specifically to the E2F1-binding site in CpG island 3 of the POLD1 promoter (Fig. 2b).

### POLD1 expression was positively correlated with E2F1 expression but inversely correlated with POLD1 promoter methylation in the replicative senescence of 2BS and WI-38 cells

**Fig. 3:**
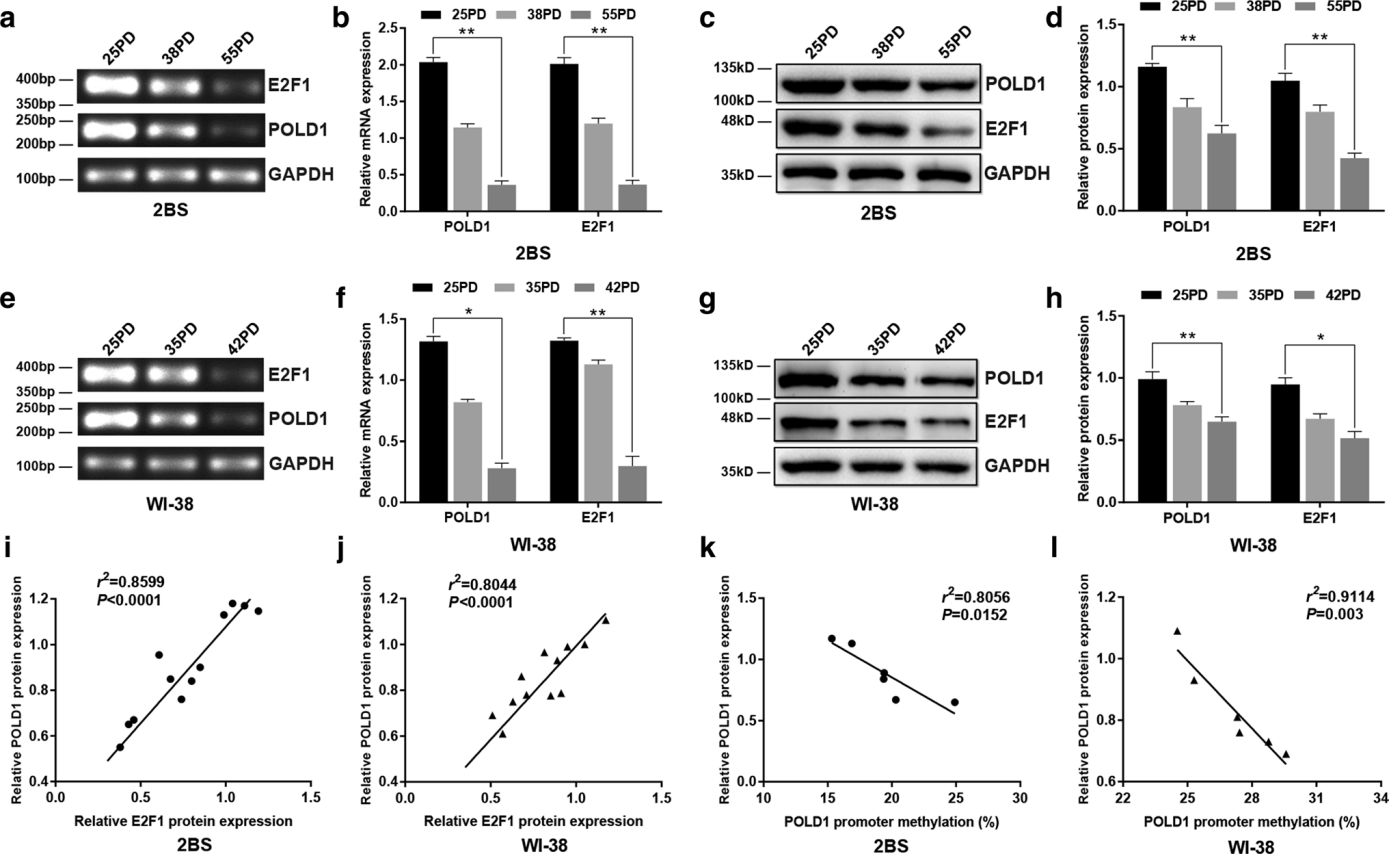
The alterations in the expression levels of POLD1 and E2F1 in replicative senescence and the relationship between the POLD1 expression level and E2F1 expression level as well as the POLD1 promoter methylation level. **a**, **b** The mRNA expression levels of POLD1 and E2F1 in different PDs of 2BS cells. **c**, **d** The protein expression levels of POLD1 and E2F1 in different PDs of 2BS cells. **e**, **f** The mRNA expression levels of POLD1 and E2F1 in different PDs of WI-38 cells. **g**, **h** The protein expression levels of POLD1 and E2F1 in different PDs of WI-38 cells. GAPDH was used for normalization of the data. Values represent the mean ± SD. The data were analyzed using one-way ANOVA, with three independent experiments in each group; *p < 0.05, **p < 0.01, vs. the young cells (25PD). **i**, **j** Correlation between the relative POLD1 protein expression level (*Y*-axis) and the relative E2F1 expression level (*X*-axis) in 2BS and WI-38 cells. Analysis for the correlation of E2F1 and POLD1 expression with Spearman’s rho method. **k**, **l** Correlation between the mean relative POLD1 expression level (*Y*-axis) and DNA methylation level (%) of the POLD1 promoter (*X*-axis) in 2BS and WI-38 cells. The correlation was determined using Pearson’s correlation coefficient

The POLD1 and E2F1 expression levels were measured by RT-PCR and western blotting in different PDs of 2BS and WI-38 cells, and the correlativity of E2F1 and POLD1 expression was calculated by means of Spearman’s rho method. The data showed that the mRNA and protein levels of POLD1 and E2F1 were both decreased in aging cells compared to young cells (Fig. 3). Moreover, the POLD1 expression level was positively correlated with the E2F1 expression level in 2BS (*n *= 12, *r*^2^= 0.8599, *p *< 0.0001, Fig. 3i) and WI-38 cells (*n *= 12, *r*^2^= 0.8044, *p *< 0.0001, Fig. 3j). These results indicated that the decreased expression of the transcription factor E2F1 could be related to the downregulation of POLD1 in replicative senescence.

Furthermore, the correlation between the methylation level of the POLD1 promoter region and the POLD1 expression level was calculated using Pearson’s correlation coefficient, and the results showed an inverse relationship between POLD1 expression and POLD1 promoter methylation in 2BS (*n *= 6, *r*^2^= 0.8056, *p *= 0.0152, Fig. 3k) and WI-38 cells (*n *= 6, *r*^2^= 0.9114, *p *= 0.003, Fig. 3l). These results suggested that the age-related increase in the POLD1 promoter methylation level, especially the age-related increase in the methylation level of CpG island 3, which binds to E2F1, could be closely related to the downregulation of POLD1 in cell aging.

### POLD1 promoter methylation silencing POLD1 promoter activity

**Fig. 4:**
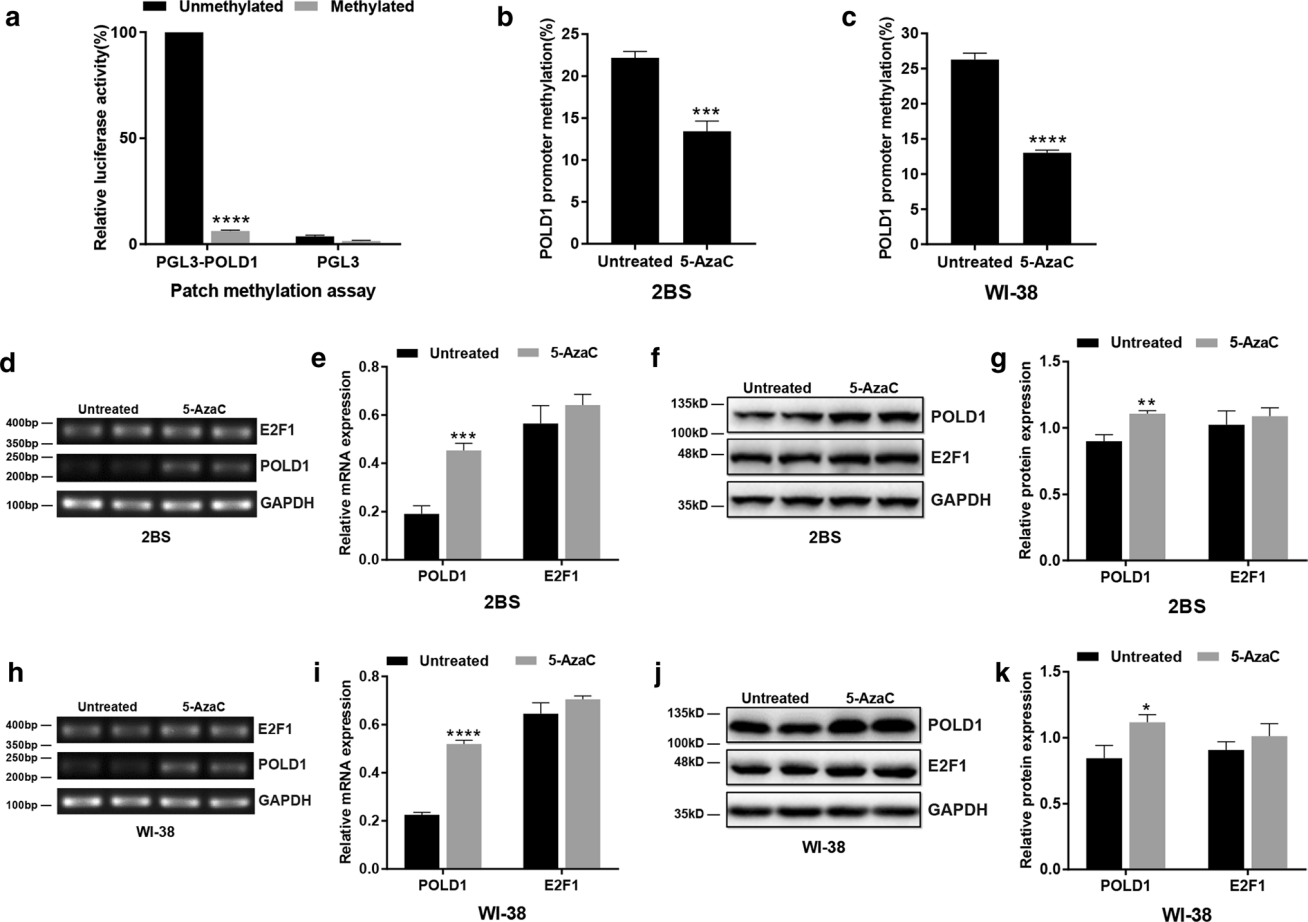
Effect of methylation on POLD1 promoter activity and effect of 5-AzaC treatment on POLD1 and E2F1 expression. **a** The POLD1 promoter activity in HEK-293T cells transfected with the unmethylated and methylated pGL3 control and pGL3-POLD1 plasmids. Comparison of luciferase activity in HEK-293T cells cotransfected with a pRL-CMV vector containing β-galactosidase and a pGL3 vector containing a luciferase gene driven by a methylated or unmethylated POLD1 promoter fragment (− 1758 to + 49 bp). After 24 h, luciferase activity in cell lysates, related to β-galactosidase activity, was determined, and relative luciferase activity was normalized to the activity of an unmethylated control pGL3 vector. The results are presented as the ratio (%) of luciferase activity to the unmethylated control. Values represent the mean ± SD, with three independent experiments per group. **b**, **c** DNA methylation status of the POLD1 promoter in 48PD 2BS and 38PD WI-38 cells after treatment with 5 μM 5-AzaC by bisulfite DNA sequencing analysis. **d**, **e** The mRNA expression levels of POLD1 and E2F1 in 48PD 2BS cells treated with 5 μM 5-AzaC for 72 h. **f**, **g** The protein expression levels of POLD1 and E2F1 in 48PD 2BS cells treated with 5 μM 5-AzaC for 72 h. **h**, **i** The mRNA expression levels of POLD1 and E2F1 in 38PD WI-38 cells treated with 5 μM 5-AzaC for 72 h. **j**, **k** The protein expression levels of POLD1 and E2F1 in 38PD WI-38 cells treated with 5 μM 5-AzaC for 72 h. GAPDH was used for normalization of the data. The data were compared with the independent samples t-test, with three independent experiments in each group; *p < 0.05, **p < 0.01, ***p < 0.005, ****p < 0.0001, vs. the untreated group

To investigate whether methylation plays a direct role in silencing POLD1 promoter activity, the ‘Patch’ methylation method was used in this study. Low luciferase activities were observed when the POLD1 promoter was methylated, indicating that methylation suppresses promoter activity (Fig. 4a).

### Effect of 5-AzaC treatment on the expression of POLD1 and E2F1 in 2BS and WI-38 cells

To illustrate the effects of demethylation reagents 5-AzaC on the expression of POLD1 and E2F1, the mRNA and protein levels of POLD1 and E2F1 were measured in 48PD 2BS and 38PD WI-38 cells treated with 5 μM 5-AzaC. The POLD1 promoter methylation levels were inhibited (Fig. 4b, c), and POLD1 expression was apparently increased (Fig. 4); however, there were no significant changes in E2F1 expression (Fig. 4). These results demonstrated that 5-AzaC can promote the expression of POLD1 but has no effect on E2F1 expression.

### E2F1 regulates the expression of POLD1

**Fig. 5:**
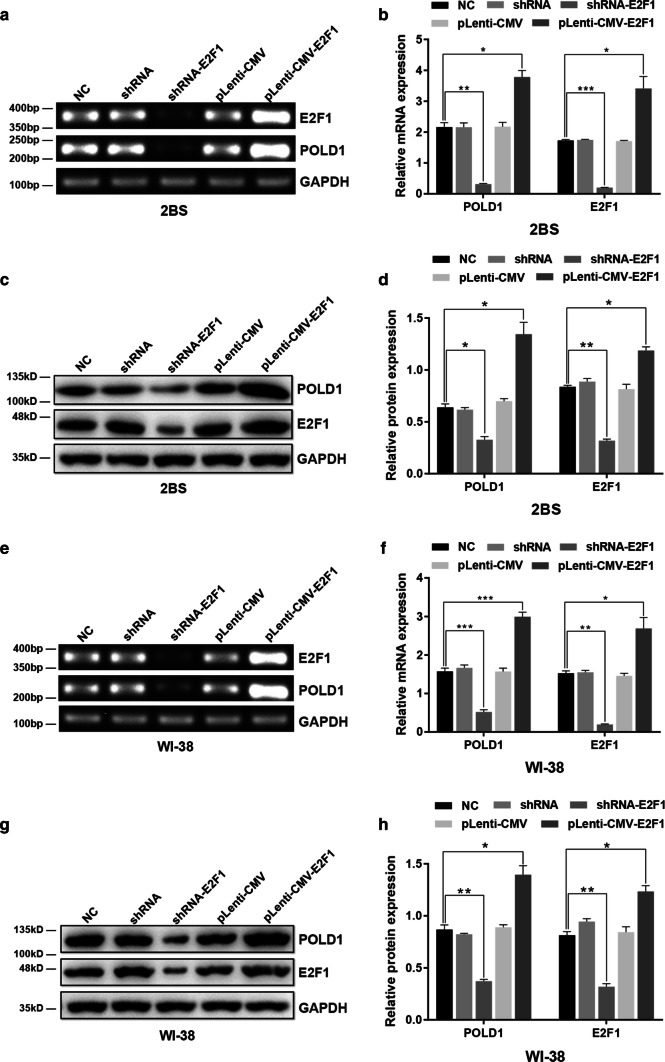
The effect of E2F1 on the expression of POLD1. 38PD 2BS and 35PD WI-38 cells were transfected with shRNA targeting E2F1 and E2F1 expression lentivirus. RT-PCR and western blotting were used to analyze the expression levels of E2F1 and POLD1. Control shRNA and pLenti-CMV were used as negative controls, and a bar graph indicates the relative expression levels of POLD1 and E2F1, normalized to GAPDH. The data were compared by one-way ANOVA, with three independent experiments in each group; *p < 0.05, **p < 0.01, ***p < 0.005, vs. the normal control (NC)

To illustrate the biological importance of E2F1 in POLD1 gene regulation, RNA interference (RNAi) and overexpression methods were used to regulate E2F1 expression in 38PD 2BS and 35PD WI-38 cells. Then, the mRNA and protein levels of POLD1 and E2F1 were examined by RT-PCR and western blot. The results showed that the expression of E2F1 was dramatically upregulated after transfection of the E2F1 eukaryotic expression lentivirus and downregulated after transfection with shRNA-E2F1 (Fig. 5).

As shown in Fig. 5, the mRNA and protein levels of POLD1 increased significantly in the cells transfected with pLenti-CMV-E2F1 compared with the cells in the other groups. However, the expression levels of POLD1 clearly decreased in the cells transfected with shRNA-E2F1. These results demonstrated that the expression level of E2F1, a key transcription factor of POLD1, was an important determinant in the regulation of POLD1 expression. Moreover, these results also indirectly confirmed that the age-related decline in E2F1 expression partly contributes to the decrease in POLD1 expression in replicative senescence.

### Synergistic effect of E2F1 and 5-AzaC on POLD1 expression

**Fig. 6:**
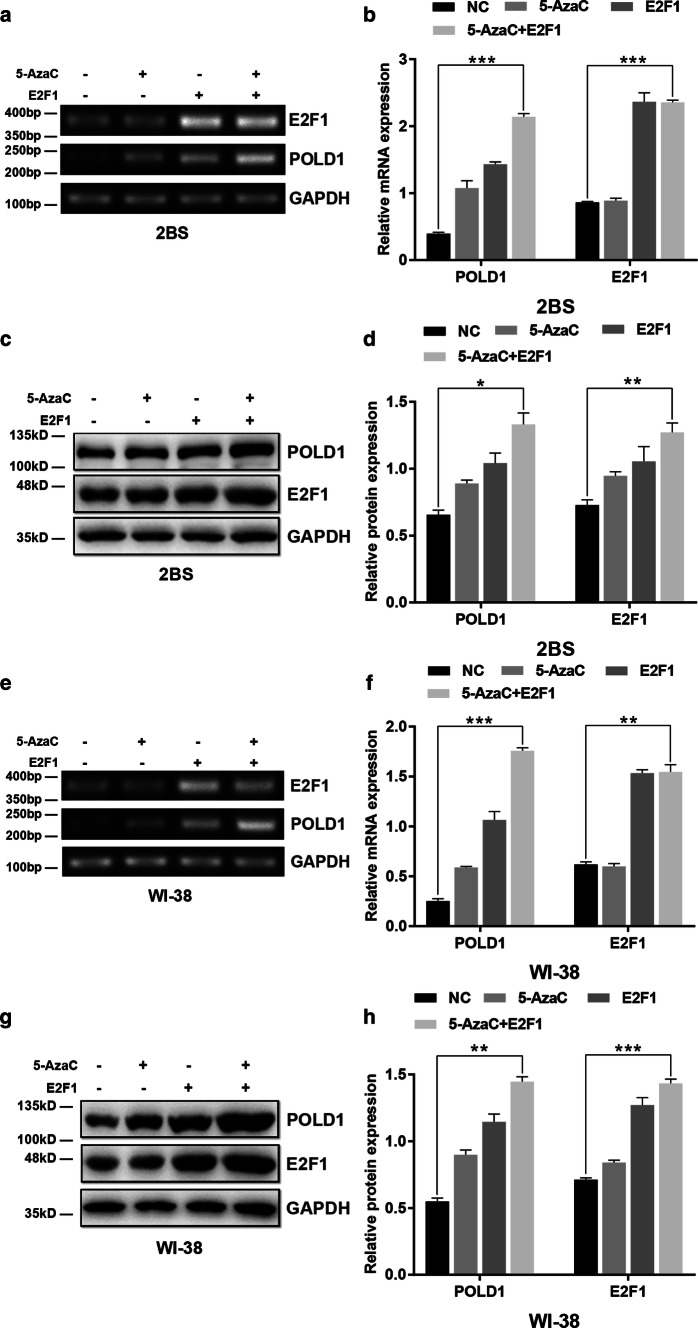
Effect of E2F1 and 5-AzaC treatment on POLD1 expression. 48PD 2BS and 38PD WI-38 cells were treated with 5 μM 5-AzaC and/or transfected with E2F1 expression lentivirus. **a**, **b** The effect of E2F1 overexpression and/or 5-AzaC treatment on the mRNA expression of POLD1 in 48PD 2BS cells. **c**, **d** The effect of E2F1 overexpression and/or 5-AzaC treatment on the protein expression of POLD1 in 48PD 2BS cells. **e**, **f** The effect of E2F1 overexpression and/or 5-AzaC treatment on the mRNA expression of POLD1 in 38PD WI-38 cells. **g**, **h** The effect of E2F1 overexpression and/or 5-AzaC treatment on the protein expression of POLD1 in 38PD WI-38 cells. GAPDH was used for normalization of the data. The data were compared by one-way ANOVA, with three independent experiments in each group; *p < 0.05, **p < 0.01, ***p < 0.005, vs. the NC

To investigate the synergistic effect of 5-AzaC and E2F1 on POLD1 expression, the levels of POLD1 were examined in 48PD 2BS and 38PD WI-38 cells treated with 5 μM 5-AzaC for 24 h and/or transfected with E2F1 expression lentivirus. As shown in Fig. 6, although the POLD1 expression level both in the cells treated with 5-AzaC and in the cells transfected with E2F1 expression lentivirus increased significantly compared to that in the control cells, the expression level of POLD1 in the cells cotreated with 5-AzaC and E2F1 overexpression lentivirus was highest among all the groups. The results strongly demonstrated that E2F1 and 5-AzaC have synergistic effects on POLD1 expression.

### The variation in the binding affinity of E2F1 for the POLD1 promoter in replicative senescence and the effects of promoter methylation and the E2F1 level on the binding affinity of E2F1 for the POLD1 promoter

**Fig. 7:**
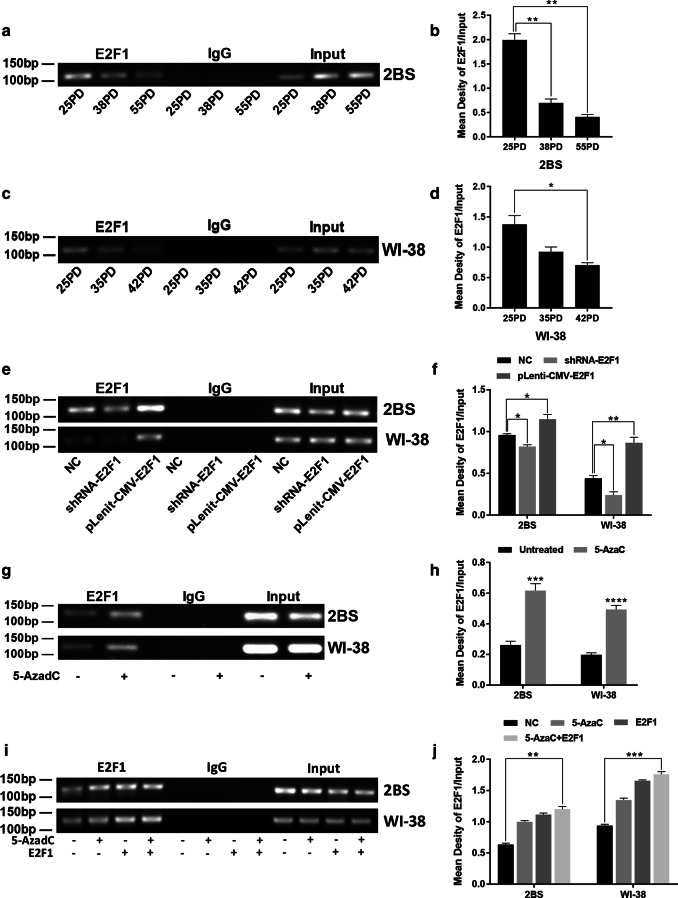
The variation in the binding affinity of E2F1 for the POLD1 promoter in replicative senescence and the effects of E2F1 and 5-AzaC treatment on the binding affinity of E2F1 for the POLD1 promoter in 2BS and WI-38 cells. Chromatin immunoprecipitation (ChIP) analysis was used to evaluate the binding affinity of E2F1 for the POLD1 promoter. **a**, **b** The binding affinity of E2F1 for the POLD1 promoter in different PDs of 2BS cells. **c**, **d** The binding affinity of E2F1 for the POLD1 promoter in different PDs of WI-38 cells. **e**, **f** The binding affinity of E2F1 for the POLD1 promoter in 48PD 2BS and 38PD WI-38 cells transfected with shRNA and expression lentivirus of E2F1. **g**, **h** The binding affinity of E2F1 for the POLD1 promoter in 48PD 2BS and 38PD WI-38 cells treated with 5 μM 5-AzaC after 72 h. **i**, **j** The binding affinity of E2F1 for the POLD1 promoter in 48PD 2BS and 38PD WI-38 cells treated with 5 μM 5-AzaC after 24 h and/or transfected with the expression lentivirus of E2F1. Densitometric analysis of the band intensity in different groups, normalized to the input. The data were compared by one-way ANOVA and the independent samples t-test, with three independent experiments in each group, *p < 0.05, **p < 0.01, ***p < 0.005, vs. the NC

To investigate alterations in the binding affinity of E2F1 for the POLD1 promoter in the replicative senescence of 2BS and WI-38 cells, ChIP assays were used. The results showed that the binding affinity of E2F1 for the POLD1 promoter was markedly decreased in replicative senescence (Fig. 7a, c), which could be directly responsible for the age-related downregulation of POLD1.

To further investigate the mechanism underlying the alteration in the binding affinity of E2F1 for the POLD1 promoter in replicative senescence, the effect of the E2F1 expression level and promoter methylation level on the binding affinity of E2F1 for the POLD1 promoter was examined in 48PD 2BS and 38PD WI-38 cells. The results showed that the binding affinity of E2F1 for the POLD1 promoter in the cells transfected with the E2F1 expression lentivirus was higher than that in the control cells, and the binding affinity in the cells transfected with shRNA-E2F1 was lower than that in the control cells (Fig. 7e). The binding affinity of E2F1 to the POLD1 promoter in the cells treated with 5 μM 5-AzaC was significantly higher than that in the untreated cells (Fig. 7g). Moreover, the affinity in the cells cotreated with 5-AzaC and pLenit-CMV-E2F1 was markedly higher than that in the cells treated with 5-AzaC or transfected with E2F1 expression lentivirus (Fig. 7i). These results strongly demonstrated that the binding affinity level of E2F1 for the POLD1 promoter is determined by the E2F1 expression level and POLD1 promoter methylation level.

These results and the results above indicated that the decrease in the binding affinity of E2F1 for the POLD1 promoter, which is attributed to an age-related decrease in the E2F1 level and increase in POLD1 promoter methylation level (especially CpG island 3), leads to a decrease in POLD1 expression in replicative senescence.

### Effects of altered E2F1 expression and DNA methylation on cell growth and proliferation, DNA synthesis, and DNA damage levels in 2BS and WI-38 cells

To investigate the effect of E2F1 and 5-AzaC on cell aging, cell growth, and proliferation, DNA synthesis and DNA damage levels in 2BS and WI-38 cells treated with 5-AzaC or transfected with pLenti-CMV-E2F1 and shRNA-E2F1 were tested using the CCK-8 assay, EdU incorporation assay and Comet assay, respectively.

**Fig. 8:**
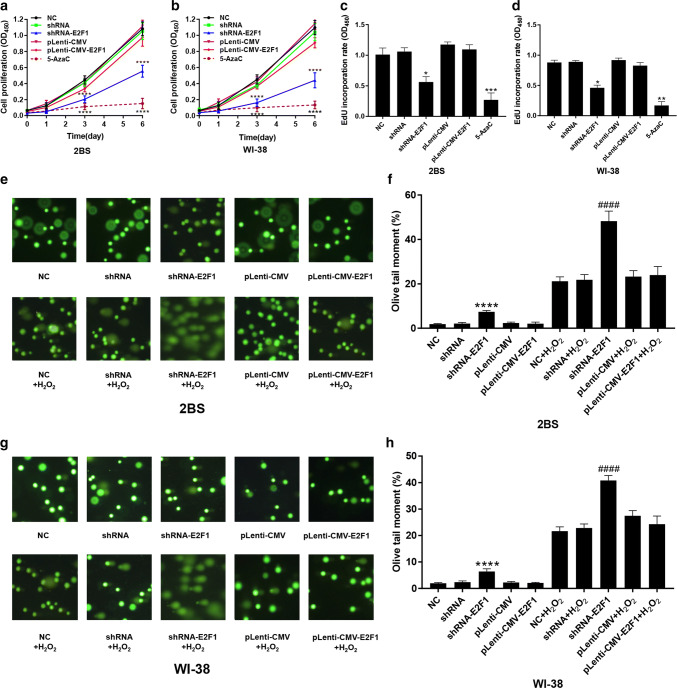
Effects of altered E2F1 expression and DNA methylation on cell growth and proliferation, DNA synthesis, and DNA damage levels in 2BS and WI-38 cells. **a**, **b** The proliferation of 48PD 2BS and 38PD WI-38 cells transfected with the indicated lentivirus or treated with 5 μM 5-AzaC was determined by CCK-8 assay. The absorbance at 450 nm was measured at 0, 1, 3, and 6 days. The data shown are the mean ± SD of the ratio for the absorbance. The data were compared by two-way ANOVA, with three independent experiments in each group; ****p < 0.001, vs. the NC. **c**, **d** Quantification of the DNA synthesis rate in 2BS and WI-38 cells transfected with the indicated lentivirus and/or treated with 5 μM 5-AzaC. An EdU incorporation assay was used. The EdU-positive cells were quantified after 48 h of treatment by the ratio of the absorbance at 450 nm. The data were compared by one-way ANOVA, with three independent experiments in each group; *p < 0.05, **p < 0.01, ***p < 0.005, vs. the NC. **e**–**h** Effects of altered E2F1 expression on DNA damage in 48PD 2BS and 38PD WI-38 cells. 2BS and WI-38 cells were transfected with the indicated lentivirus. The cells were treated with 100 μM H_2_O_2_ for 5 min in the dark. A comet assay was then performed, and images were acquired using fluorescence to show DNA fragment migration patterns (magnification, × 100). The olive tail moments determined by CAPS software (*n* = 50). The data were compared by one-way ANOVA; ****p < 0.001, vs. the NC and ^####^*p* < 0.001, vs. NC + H_2_O_2_

The results showed that the growth and proliferation of the cells transfected with shRNA-E2F1 decreased significantly compared to that of the cells in the other groups (Fig. 8a, b). Additionally, there was no significant difference in the growth and proliferation of the cells overexpressing E2F1 compared to the control cells. However, the growth and proliferation of the cells treated with 5 μM 5-AzaC was lowest among the cells in all groups (Fig. 8a, b).

The results also showed that the EdU incorporation rate of the cells transfected with shRNA-E2F1 was lower than that of the control cells (Fig. 8c, d). These results indicated that E2F1 is important in cell proliferation. The EdU incorporation rate of the cells treated with 5-AzaC was the lowest among the cells in all groups (Fig. 8c, d). These results were consistent with the results of the CCK-8 assay, which indicated that cell growth and proliferation were inhibited by 5-AzaC.

The comet assay is a simple method for measuring DNA strand breaks in eukaryotic cells, and the olive tail moment (%) represents the breakage levels of DNA [17]. The results showed that the DNA damage levels of the cells in the normal control, shRNA, pLenti-CMV and pLenti-CMV-E2F1 groups were fairly low after treatment with H_2_O_2_. However, the DNA damage level in the cells transfected with shRNA-E2F1 increased significantly, even when the cells were not treated with H_2_O_2_ (Fig. 8e, g). These results indicated that E2F1 is important in DNA damage repair.

### The methylation status of the POLD1 promoter, the expression levels of POLD1 and E2F1, and the binding affinity of E2F1 for the POLD1 promoter in the lymphocytes of healthy people of different ages

**Fig. 9:**
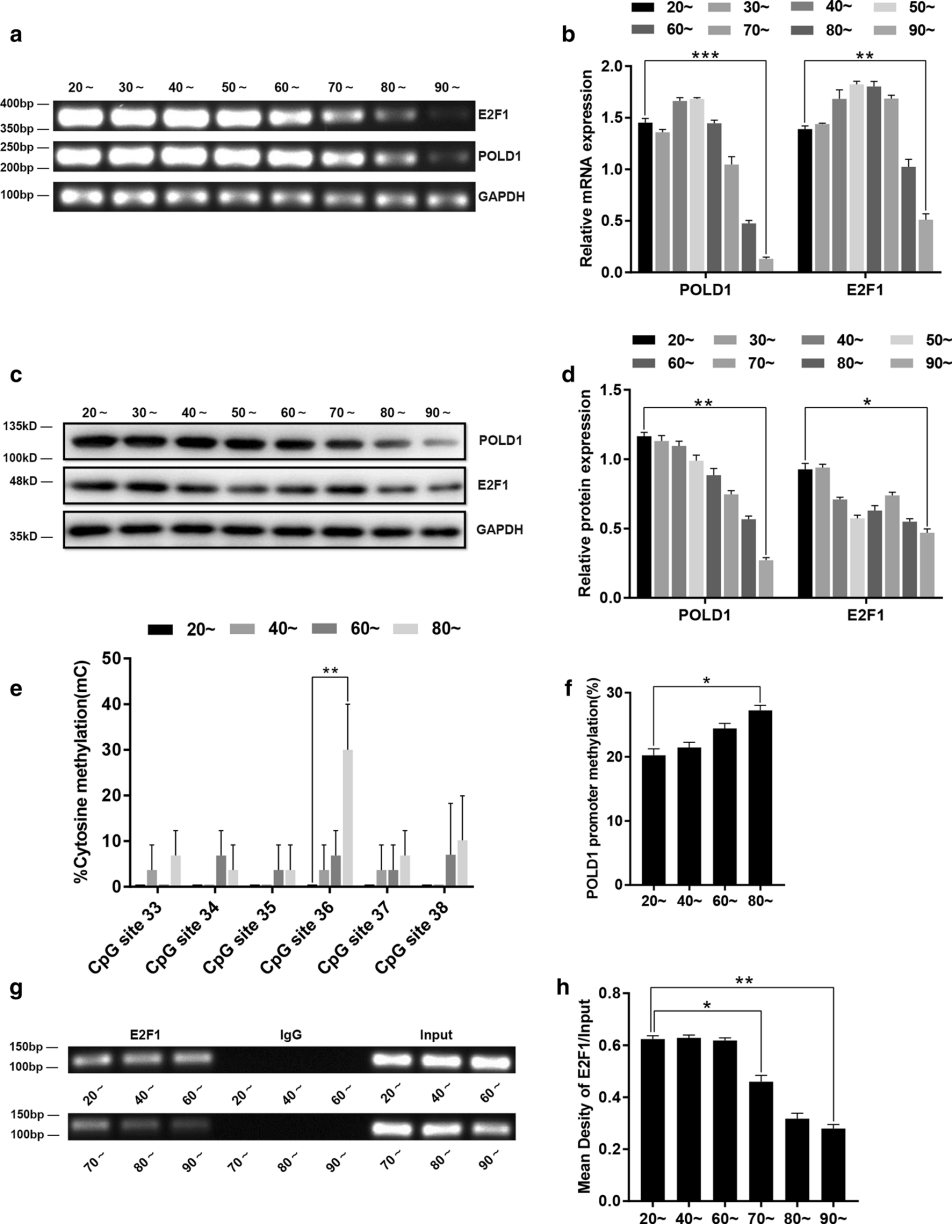
The methylation status of the POLD1 promoter, the expression levels of POLD1 and E2F1 and the binding affinity of E2F1 to the POLD1 promoter in lymphocytes from healthy people of different ages (*n* = 14 in 20 ~ years to 70 ~ years group, *n* = 8 in 80 ~ years and 90 ~ years group). **a**, **b** The mRNA expression levels of POLD1 and E2F1 in lymphocytes from healthy people of different ages. GAPDH was used for normalization of the data. Values represent the mean ± SD. **c**, **d** The protein expression levels of POLD1 and E2F1 in the lymphocytes of healthy people of different ages. GAPDH was used for normalization of the data. Values represent the mean ± SD. **e** The percentage of cytosine methylation of CpG site 33–38 of CpG island 3 in the lymphocytes of healthy people of different ages (*n* = 3 in each group), as measured by bisulfite DNA sequencing analysis. **f** The methylation levels of the POLD1 promoter in the lymphocytes of healthy people of different ages (*n* = 3 in each group). **g**, **h** The binding affinity of E2F1 for the POLD1 promoter in the lymphocytes from healthy people of different ages. Densitometric analysis of the band intensity in different groups, normalized to the input. The data were compared using one-way ANOVA, with three independent experiments in each group; *p < 0.05, **p < 0.01, *** *p *< 0.005, vs. the 20 ~ years group

To investigate the profile of E2F1-mediated regulation of POLD1 in healthy people, the methylation levels of the POLD1 promoter, the expression levels of POLD1 and E2F1, and the binding affinity of E2F1 for the POLD1 promoter were measured in the lymphocytes of healthy people of different ages. The results showed that the levels of POLD1 and E2F1 decreased gradually in the lymphocytes as the cells aged; this was especially notable for the POLD1 level, which was remarkably reduced in the lymphocytes of > 80-year-old healthy people (Fig. 9a, c). The POLD1 promoter methylation level increased notably as the ages of the donors increased and also had a unique methylation change at a single site, CpG 36, in CpG island 3 (Fig. 9e, f). Moreover, a significant decrease in the E2F1 binding affinity was observed in the lymphocytes of people over 70 years of age (Fig. 9g), though there was no significant difference in the E2F1 binding affinity in the lymphocytes derived from people who were 20–60 years of age (Fig. 9g).

These results indicated that the decline in POLD1 expression could also be due to the age-related attenuation of the E2F1 binding affinity, which could be caused by age-related decrease in the E2F1 level and improvement of POLD1 promoter methylation. Additionally, the above results suggested that POLD1, E2F1, the E2F1 binding affinity to the POLD1 promoter, and the POLD1 promoter methylation level have the potential to be aging biomarkers.

## Discussion

Cellular senescence is defined as the entry of cells into a state of irreversible growth arrest after serial cultivation [18]. Aging cells are characterized by a decline in their proliferation ability, a slow-down of the cell cycle, a limited telomeric length, evident alterations of aging-related genes, and the appearance of aging phenotypes [19]. The senescent characteristics above can be attributable to a decline in DNA synthesis, increased DNA injury, and a reduced DNA damage repair ability with aging [20]. Thus, DNA polymerase delta (Pol δ), which is responsible for DNA synthesis and DNA damage repair, can play a very important role in replicative senescence [21].

DNA pol δ consists of four subunits (p125, p50, p66, p12), of which p125 is the catalytic subunit encoded by the POLD1 gene [22]. Previous studies showed that the expression of POLD1 is downregulated with aging [6]. The reduced POLD1 level induces a decrease in cell proliferation, a delay in the cell cycle, a decline in DNA synthesis, and a remarkable increase in the DNA breakage level under oxidative stress [23], indicating that the age-related downregulation of POLD1 may be the ‘source event’ of the aging process. However, the mechanisms of the age-related decrease in POLD1 expression have not been elucidated.

DNA methylation is a process in which methyl groups are added to DNA molecules. Methylation can regulate the activity of the DNA segment without altering its sequence [24]. On the one hand, the entire genome has been found to progressively lose 5-mC in cultured cells [25]. This loss may result in aging-associated gene expression, which has been proposed as an important factor in cellular senescence [26]. On the other hand, age-dependent hypermethylation in specific genes may inhibit proliferation-associated gene expression [27].

In this study, the four CpG islands in the POLD1 promoter were first found in 2BS and WI-38 cells; CpG island 1 (109 bp) was located in the − 1878 to − 1770 region, CpG island 2 (102 bp) was located in the − 767 to − 666 region, CpG island 3 (504 bp) was located in the − 408 to + 95 region and CpG islands 4 (100 bp) was located the + 147 to + 246 region of the POLD1 promoter. The methylation status of the POLD1 promoter and the four CpG islands was measured throughout the life spans of the 2BS and WI-38 cells and in the lymphocytes of healthy people of different ages. The results showed that the DNA methylation status of the POLD1 promoter region increased significantly during the aging process (Figs. 1c, d, 9f), especially at a single site, CpG 36, in CpG island 3 (Figs. 1g, h, 9e). POLD1 expression was inversely correlated with its promoter methylation status (Fig. 3k, l). Furthermore, POLD1 promoter activity was suppressed by promoter methylation in a ‘Patch’ methylation assay (Fig. 4a).

In a previous study, E2F1 was demonstrated to be a growth-regulatory gene, and E2F1 plays an important role in cell proliferation and cell apoptosis as a crucial regulator of the cell cycle [28]. For the first time, this study proved that E2F1 binds specifically to the E2F1-binding site in CpG island 3 of the POLD1 promoter (Fig. 2b), and the binding affinity of E2F1 for CpG island 3 of the POLD1 promoter was verified to be attenuated by aging in vitro and in vivo (Figs. 7a, c, 9g). Moreover, the age-related decrease in E2F1 (Fig. 3c, g) and the positive relationship between the E2F1 level and POLD1 expression level were also observed (Fig. 3i, j).

The results above indicated that the decrease in the binding affinity of E2F1 for the POLD1 promoter might be the direct cause of POLD1 downregulation in replicative senescence. Additionally, the parallel changes in terms of the decreased E2F1 expression, the hypermethylation of CpG island 3 and the decreased binding affinity of E2F1 for the POLD1 promoter suggest that the E2F1 binding affinity to the POLD1 promoter might be influenced by E2F1 expression and the methylation status of CpG island 3 in cellular senescence.

To ascertain this supposition, E2F1 expression was knocked down or upregulated in this study (Fig. 5). Compared to the normal control, both the E2F1 binding affinity to the POLD1 promoter and POLD1 expression were confirmed to be enhanced with higher E2F1 expression and weakened with lower E2F1 expression (Fig. 7e). These results demonstrated that the E2F1 binding affinity to the POLD1 promoter was positively regulated by E2F1 expression and indicated that the decrease in E2F1 expression contributed to the decreased binding affinity of E2F1 to the POLD1 promoter in replicative senescence.

As the traditional view, transcription factors (TFs) usually bind to nonmethylated DNA motifs in open chromatin regions [29]. The methylation level of CpG island 3, the site of E2F1 binding, was lower in young cells and higher in aged cells. To further investigate how CpG island 3 methylation affects E2F1 binding affinity, the DNA methylation inhibitor 5-AzaC was used to demethylate the POLD1 promoter. The results showed that both the binding affinity of E2F1 to the POLD1 promoter and POLD1 expression increased significantly in the 5-AzaC-treated cells compared to the untreated group (Figs. 4f, j, 7g). These findings demonstrated that the binding affinity of E2F1 to the POLD1 promoter is negatively regulated by POLD1 promoter methylation and indicated that the age-related increase in CpG island 3 methylation also contributes to the decrease in the E2F1 binding affinity to the POLD1 promoter in replicative senescence.

The synergistic effect of E2F1 upregulation and POLD1 promoter demethylation on the binding affinity of E2F1 for the POLD1 promoter, as well as POLD1 expression, was further observed. The results showed that both the binding affinity of E2F1 to the POLD1 promoter and POLD1 expression were significantly higher in the cells treated with E2F1-overexpression/5-AzaC than in the cells treated with E2F1 overexpression or 5-AzaC alone (Figs. 6c, g, 7i). These findings suggest a synergistic effect for the age-associated decline of E2F1 levels and the elevation in CpG island 3 methylation on the binding affinity of E2F1 for the POLD1 promoter as well as POLD1 expression in replicative senescence.

**Fig. 10:**
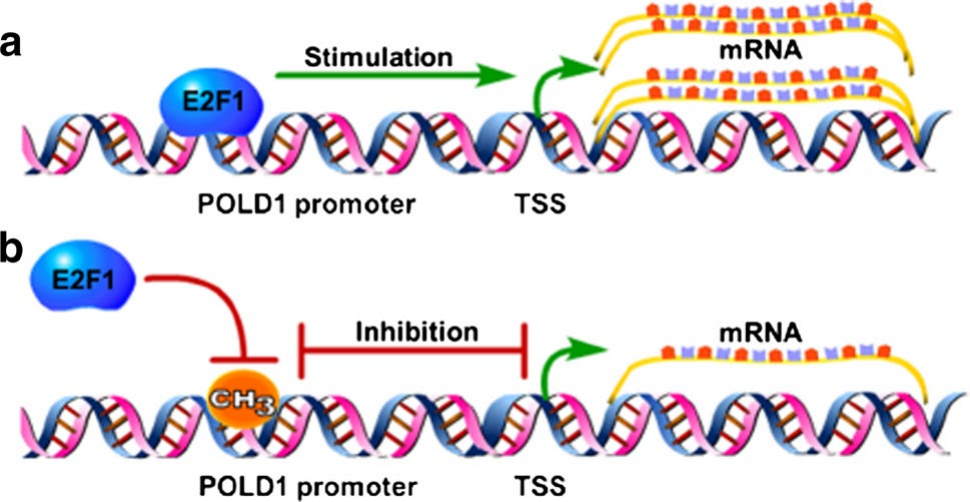
The regulatory mechanism of E2F1 and DNA methylation in POLD1 expression. **a** E2F1 combines with the POLD1 promoter and stimulates POLD1 expression. **b** DNA methylation blocks the binding of E2F1 to the POLD1 promoter and inhibits POLD1 expression. TSS: transcription start site

The above results fully support the conclusion of this study (Fig. 10) that the attenuation of the binding affinity of E2F1 for the POLD1 promoter, mediated by an age-related decline in E2F1 and an increase in CpG island 3 methylation, downregulates POLD1 expression in replicative senescence.

To verify the effect of the E2F1 expression level and demethylation reagents 5-AzaC on cellular senescence, the cell proliferation, DNA synthesis rate, and DNA damage repair ability were measured in the cells treated with 5-AzaC or transfected with pLenti-CMV-E2F1 or shRNA-E2F1. Striking decreases in cell proliferation and the EdU incorporation rate and a notable increase in DNA damage under oxidative stress were observed in the cells transfected with shRNA-E2F1 compared with the control cells (Fig. 8). However, there were no significant alterations in the cells transfected with pLenti-CMV-E2F1 compared to the control cells. These results indicated that E2F1 is important in cell proliferation and DNA damage repair. However, cell proliferation and the EdU incorporation rate decreased significantly in the cells treated with 5-AzaC, which could be due to complex changes in multiple genes that are affected by global DNA demethylation. In fact, a previous study [30] about the effect of the inhibition of DNA methyltransferase on cell growth, provides support for this conjecture.

Although the functions of other transcription factors in the POLD1 promoter need to be further clarified, we propose the following POLD1 theory of replicative senescence based on this study: An age-related decline in POLD1 expression, induced by altered expression of transcription factors and an increase in POLD1 promoter methylation, promotes replicative senescence by reducing cell proliferation, DNA synthesis and DNA damage repair ability, among other processes.

## Abbreviations

Pol δ: DNA polymerases delta
TF: Transcription factor
TFBS: Transcription factor binding site
RT-PCR: Reverse transcriptional polymerase chain reaction
ChIP: Chromatin immunoprecipitation
PD: Population doubling
FBS: Fetal bovine serum
PBS: Phosphate-buffered saline
PBMC: Peripheral blood mononuclear cells
HRP: Horseradish peroxidase
BSP: Bisulfite sequencing
shRNA: Short hairpin RNA
PMSF: Phenylmethanesulfonyl fluoride
5-AzaC: 5′-Aza-2′-deoxycytidine
CCK-8: Cell counting kit-8
EdU: 5-Ethynyl-2′-deoxyuridine
OTM: Olive tail moment
TSS: Transcription start site
